# Hormone-sensing cells require Wip1 for paracrine stimulation in normal and premalignant mammary epithelium

**DOI:** 10.1186/bcr3381

**Published:** 2013-01-31

**Authors:** Gerard A Tarulli, Duvini De Silva, Victor Ho, Kamini Kunasegaran, Kakaly Ghosh, Bryan C Tan, Dmitry V Bulavin, Alexandra M Pietersen

**Affiliations:** 1 Laboratory of Mammary Gland Biology, National Cancer Centre Singapore, 11 Hospital Dr, 169610, Singapore; 2 Program in Cancer & Stem Cell Biology, Duke-NUS Graduate Medical School, 8 College Rd, 169857, Singapore; 3 Institute of Molecular & Cell Biology, 61 Biopolis Dr, Proteos 138673, Singapore; 4 Department of Physiology, National University of Singapore, 21 Lower Kent Ridge Rd, 119077, Singapore

## Abstract

**Introduction:**

The molecular circuitry of different cell types dictates their normal function as well as their response to oncogene activation. For instance, mice lacking the Wip1 phosphatase (also known as PPM1D; protein phosphatase magnesium-dependent 1D) have a delay in HER2/neu (human epidermal growth factor 2), but not Wnt1-induced mammary tumor formation. This suggests a cell type-specific reliance on Wip1 for tumorigenesis, because alveolar progenitor cells are the likely target for transformation in the MMTV(mouse mammary tumor virus)-*neu *but not MMTV-*wnt1 *breast cancer model.

**Methods:**

In this study, we used the *Wip1*-knockout mouse to identify the cell types that are dependent on *Wip1 *expression and therefore may be involved in the early stages of HER2/neu-induced tumorigenesis.

**Results:**

We found that alveolar development during pregnancy was reduced in *Wip1*-knockout mice; however, this was not attributable to changes in alveolar cells themselves. Unexpectedly, Wip1 allows steroid hormone-receptor-positive cells but not alveolar progenitors to activate STAT5 (signal transducer and activator of transcription 5) in the virgin state. In the absence of Wip1, hormone-receptor-positive cells have significantly reduced transcription of *RANKL *(receptor activator of nuclear factor kappa-B ligand) and *IGF2 *(insulin-like growth factor 2), paracrine stimulators of alveolar development. In the MMTV-*neu *model, HER2/neu activates STAT5 in alveolar progenitor cells independent of Wip1, but HER2/neu does not override the defect in STAT5 activation in Wip1-deficient hormone-sensing cells, and paracrine stimulation remains attenuated. Moreover, ERK (extracellular signal-regulated kinase) activation by HER2/neu in hormone-sensing cells is also Wip1 dependent.

**Conclusions:**

We identified Wip1 as a potentiator of prolactin and HER2/neu signaling strictly in the molecular context of hormone-sensing cells. Furthermore, our findings highlight that hormone-sensing cells convert not only estrogen and progesterone but also prolactin signals into paracrine instructions for mammary gland development. The instructive role of hormone-sensing cells in premalignant development suggests targeting Wip1 or prolactin signaling as an orthogonal strategy for inhibiting breast cancer development or relapse.

## Introduction

Breast cancer consists of multiple subtypes, and it has been postulated that the difference between subtypes arises in part from the type of mammary epithelial cell that transforms [1,2]. The molecular circuitry of a particular cell type determines how it responds to activation of a signaling pathway and likely dictates the sensitivity of that cell to particular oncogenic mutations [3]. For instance, *Wip1*-knockout mice have a delay in tumorigenesis in the MMTV-*neu *model of breast cancer, but not in the MMTV-*wnt1 *model [4]. *Wip1 *is overexpressed in ~20% of human breast cancer cases, which belong mostly to the luminal and HER2^+ ^subtypes [5]. Together, this suggests that the target cells for transformation by HER2/neu activation are dependent on Wip1, whereas those that can be transformed by Wnt1 are not.

Wip1 is a serine/threonine phosphatase of the PP2C (protein phosphatase 2C) family, and its oncogenic function has been attributed to, for instance, its role as a negative regulator of p53 by dephosphorylating key members of DNA-damage signaling, including ATM, Chk2, and p53 itself [6]. In addition, Wip1 dephosphorylates and thereby inactivates the stress kinase p38MAPK, and inhibition of p38MAPK in Wip1-knockout mice partially restored sensitivity to MMTV-*neu*-induced tumorigenesis [7]. In this study, we examined the role of Wip1 in mammary epithelium to identify the cell types that are dependent on Wip1 activity and therefore may be involved in the early stages of HER2/neu-induced tumorigenesis.

Mammary epithelium consists of an outer basal layer of mainly contractile myoepithelial cells and an inner luminal layer that contains both steroid-receptor-positive cells and steroid-receptor-negative cells in a spatially ordered pattern [8]. Mammary gland development during puberty is orchestrated by the steroid sex hormones estrogen and progesterone, which trigger proliferation indirectly in steroid-receptor-negative cells through paracrine factors produced by steroid-receptor-positive cells. Interestingly, steroid-receptor-positive cells act mainly as a conduit for proliferative signals, as they rarely divide themselves [9,10]. The luminal steroid-receptor-negative cells contain different progenitor subsets, including alveolar progenitor cells that are primed for milk production [11-13]. During the initial phase of pregnancy, progesterone, together with the peptide-hormone prolactin, triggers a massive expansion of the alveolar cell population in a process termed lobulo-alveologenesis, followed by terminal differentiation of the alveolar cells later in pregnancy [14,15]. Both processes are strictly dependent on prolactin signaling, as any mutant in the prolactin receptor-JAK2-STAT5 signaling cascade has a defect in alveolar development [16-18], and even after alveologenesis has been completed, lactation remains dependent on STAT5 expression [19]. Activation of the prolactin receptor results in activation of the associated JAK2, which subsequently phosphorylates STAT5, allowing STAT5 to translocate to the nucleus and activate gene transcription [20]. STAT5 directly binds to the promoter of milk genes, suggesting that in mammary epithelium, alveolar cells are the principal responders to prolactin [21].

The cells most likely to be sensitive to transformation by Wnt1 are stem or progenitor cells that are part of the basal layer [22,23]. In contrast, compelling evidence suggests that the target cell for transformation in the MMTV-*neu *model belongs to the alveolar lineage. Whey acidic protein (WAP) is one of the components of milk that is expressed late during alveolar differentiation. Lineage tracing with a WAP-promoter-driven Cre recombinase, together with a Rosa-lox-stop-lox-*LacZ *reporter, showed that early lesions in MMTV-*neu *mammary glands are all LacZ-positive, indicating that these cells expressed milk genes at some point [24]. These LacZ-marked cells are also referred to as parity-identified mammary epithelial cells (PI-MECs) or lobule-restricted progenitors [25]. Strikingly, mice with a cyclin D1 point mutation generate normal mammary ducts, but no PI-MECs, and are completely resistant to MMTV-*neu *tumorigenesis [26].

In line with the presumptive alveolar origin of HER2/neu-driven tumors and the attenuation of tumorigenesis in the absence of Wip1, we found delayed alveolar development during pregnancy in *Wip1*-knockout mammary glands. Unexpectedly, we identify a role for Wip1 in steroid-receptor-positive cells rather than adjacent alveolar progenitor cells. We show that in the virgin state, only steroid-receptor-positive cells activate STAT5, and this is strictly dependent on Wip1. Unlike alveolar cells that transcribe milk genes after STAT5 activation, hormone-sensing cells transcribe paracrine stimulators of alveolar proliferation (*RANKL *and *IGF2*), elucidating a role for steroid-receptor-positive cells in the growth-promoting rather than differentiation-inducing effects of prolactin. MMTV-*neu *tumors are estrogen-receptor negative but we show that before tumor formation, ERK activation by HER2/neu is most pronounced in steroid-receptor-positive cells, and this is dependent on Wip1. Finally, in virgin *Wip1*-knockout mice, HER2/neu activates STAT5 in alveolar progenitors but not steroid-receptor-positive cells, and paracrine signaling remains attenuated. This suggests that the target cells for transformation in the MMTV-*neu *model rely on Wip1-dependent signaling in neighboring cells, highlighting the instructive role of hormone-sensing cells in early pregnancy and premalignant development.

## Materials and methods

### Mice

*Wip1 *KO (*Ppm1d^-/-^*) mice were previously described [27] (129Sv-C57BL/6-FVB background). We observed no difference between *Wip1 *wild-type or heterozygote animals in the context of alveolar development, STAT5 activation or qPCR data, and therefore the "wild-type" control groups presented here consist of a mixture of wild-type and heterozygote animals. MMTV-*neu *mice used for this study (and [4,26]) express the activated rat *ErbB2 *(*c-neu*) oncogene under control of the mouse mammary tumor virus promoter (strain TG.NK) [28] and were purchased from the Jackson Laboratory (Jax#5038, FVB background). All animal protocols were approved by the SingHealth Institute Animal Care and Use Committee.

### Timed mating and carmine staining of whole-mounted mammary glands

Female mice were placed in the cage of a male after 5 PM and checked for vaginal plugs at 9 AM the following morning (Day 0). Mice were killed by carbon dioxide inhalation and one number 3 (thoracic) gland was fixed in methacarn (60% methanol, 30% chloroform, 10% acetic acid) for 24 hours. Subsequently, the gland was placed in 70% ethanol for 24 hours, and then immersed in 0.2% carmine (Sigma C1022, St. Louis, MO, USA)/0.5% aluminum potassium sulfate (Sigma-Aldrich #23,708-6, St. Louis, MO, USA) stain for 18 hours. Next, glands were transferred to 70%, 90%, and 100% ethanol for 1 hour each, followed by 100% ethanol for 18 hours. Finally, glands were transferred to methyl salicylate (Sigma M2047, St. Louis, MO, USA) for visualization and photography with an Olympus SZX12 microscope.

### Isolation of primary mammary epithelial cells

Mammary epithelial cells were isolated [29], with minor modifications. Mice were killed by carbon dioxide inhalation and the number 4 (inguinal) and 5 mammary glands were excised after removal of mammary lymph nodes. Glands were chopped 3 times by using a McIlwain tissue chopper (Mickle Laboratory Engineering, Guildford, UK) on the finest setting, with a 90-degree rotation of the base plate between each round of chopping. Chopped glands from one animal were then placed in 10 ml digestion mix containing 3 mg/ml of collagenase A (Roche 11088793001, Mannheim, Germany) and 0.67 mg/ml trypsin (Becton Dickinson (BD) 215240, Sparks, MD, USA) at 37°C for 45 minutes with agitation every 15 minutes. Digested glands were subsequently centrifuged at 1,300 rpm (340 rcf) for 6 minutes at 4°C, and the fat layer and supernatant removed. The pellet (containing mammary epithelial organoids) was resuspended in 10 ml of L15 media (Sigma L1518, St. Louis, MO, USA) containing 6% fetal calf serum (Hyclone SV30160.03, Cramlington, UK) and centrifuged at 1,500 rpm (453 rcf) at room temperature. Supernatant was removed, and the pellet was resuspended in 5 ml of red blood cell lysis buffer (Sigma R7757, St. Louis, MO, USA) and incubated at room temperature for 5 minutes before centrifugation at 1,500 rpm for 5 minutes at 4°C. From this point, all centrifugation steps were performed at 1,500 rpm at 4°C. Pellet was then resuspended in DMEM +10% FCS and incubated for 30 minutes at 37°C in a T75 flask to allow the selective adherence of fibroblasts. Media containing organoids were collected and centrifuged. Supernatant was removed, and organoids were resuspended in L15 + 6% FCS (L15+) and kept overnight at 4°C. The next day, organoids were pelleted, washed twice in Ca^2+^/Mg^2+^-free PBS/0.02% wt/vol EDTA and incubated in 2 ml of Joklik MEM (Sigma M8028, St. Louis, MO, USA) for 15 minutes at 37°C. Organoids were centrifuged and resuspended in 2 ml of 0.25% trypsin-0.04% EDTA solution (Gibco 25200, Grand Island, NY, USA) and placed at 37°C for 2 minutes to generate single cells. Next, 5 ml of 5 μg/ml DNase I (type II) in serum-free L15 (Sigma D4527, St. Louis, MO, USA) was added for a further 5 minutes at 37°C to disperse cellular clumps. Then, 7 ml of L15+ was added (henceforth, all resuspensions were performed by using L15+), and the cell solution was passed through a 40-μm cell strainer (BD 352340, Sparks, MD, USA). The resultant single cells were pelleted, resuspended in L15+, and counted by using trypan blue and a hemocytometer. Cells were brought to a concentration of 1 × 10^6^/ml and kept on ice.

### Cell labeling, flow-cytometric analysis, and fluorescence-activated cell sorting

Fluorochrome-conjugated antibodies were titrated on primary mammary epithelial cells to ensure maximal positive-to-background fluorescence ratio (see Additional file 1). Anti-mouse and/or anti-rat compensation beads (BD 552843 and 552845, respectively) were used for single-stain antibody controls. Compensation controls also included two cellular samples: unstained cells and cells with DAPI (Sigma D8417, St. Louis, MO, USA). Cells were incubated with antibodies on ice for 45 minutes with agitation each 15 minutes. Samples were then washed with twice the sample volume and resuspended in L15+ containing 200 ng/ml of DAPI, except non-DAPI compensation controls. All multiple-labeled samples were gated on FSC-A versus SSC-A and doublet discrimination (FSC-H versus FSC-W and SSC-H versus SSC-W) and DAPI negativity (see Additional file 2). Samples contained anti-CD45 to exclude lymphocytes from analysis. Cells were analyzed and sorted on a BD FACS-Aria II containing 355 nm UV, 488 nm blue, 561 nm yellow-green, and 633 nm red lasers. Sorting for culture or *in vivo *assays was performed into L15+.

### Generation of cDNA by direct reverse transcription and qPCR analysis

For analysis of transcript levels by quantitative polymerase chain reaction (qPCR), cells were sorted directly into lysis buffer (10 IU RNase inhibitor (Invitrogen 10777, Carlsbad, CA, USA), 2 m*M *DTT, 0.15% Tween-20 (Biorad) in 12 μl of nuclease-free water) in PCR tubes. Then 500 cells were sorted into each tube (making approximately 14 μl total volume). Reverse transcription was performed by using Superscript VILO (Invitrogen 11754, Carlsbad, CA, USA), as per manufacturer's protocol. Primers were designed that span introns to exclude the detection of genomic DNA and selected for optimal melt curve and amplification profiles (for primer sequences (see Additional file 3). qPCR was performed by using SSo Fast Evagreen supermix reagent (Biorad 172-500, Hercules, CA, USA) as per manufacturer's protocol. Per subpopulation, two to three tubes were assayed, normalized with *HPRT *(validated to be consistent between groups), averaged, and compared with matched WT samples according to the delta-delta c(t) method. The relative values from three to five sets of mice were assessed with paired *t *test for statistical significance.

### Mammary gland transplantation and immunofluorescence

The number 4 and 5 mammary glands were harvested from donor mice, and the mammary glands digested and sorted, as outlined earlier. Then 25,000 bulk epithelial cells were injected into cleared number 4 fat pads of 21-day-old WT-recipient mice and allowed to engraft for 8 weeks. Glands were then harvested, fixed, and stained with carmine alum, as outlined earlier. After whole-mount analysis, glands were removed from methyl salicylate and washed 5 times for 1 hour in 100% EtOH before immersion in xylene for 2 × 1 hour. Tissue was then embedded in paraffin and processed for immunofluorescence.

### Confocal immunofluorescence

Fresh number 3 mammary glands were fixed for 18 hours in 4% buffered formaldehyde (ICM Pharma, Singapore), processed, and embedded in paraffin wax. The 5-μm sections were cut and adhered to Superfrost Plus-coated slides (Menzel-Glaser J1800AMNZ, Braunschweig, Germany) overnight at 37°C. Sections were deparaffinized in xylene (2 × 5 minutes) and 100% ethanol (2 × 5 minutes), before rehydration in graded ethanol (90%, 2 × 5 minutes; 70%, 2 × 5 minutes) and immersion in distilled H_2_O. Antigen retrieval was performed in 600 ml of 1 m*M *disodium-EDTA by heating in a microwave on high for 5 minutes, on 30% power for an additional 5 minutes, and then cooled at room temperature for 1 hour. Slides were immersed in distilled H_2_O and washed in PBS for 5 minutes. Sections were encircled with a wax pen and primary antibody diluted in PBS (for dilutions and suppliers, see Additional file 1) + 10% normal serum from the species in which the secondary antibody was raised, was applied and incubated at 4°C overnight. Sections were washed in PBS (2 × 5 minutes) before the addition of secondary antibody (in PBS + 10% normal serum), for 30 minutes at room temperature. Sections were washed in PBS (2 × 5 minutes) before the addition of DAPI (1 μg/ml) for 2 minutes at room temperature. Sections were then washed in PBS and mounted in Vectashield fluorescence mounting media (Vector Laboratories H-1000, Burlingame, CA, USA) for visualization. Images were acquired on a Zeiss 710 confocal microscope with a pinhole aperture of 1 Airy unit. Negative controls can be found in Additional file 4. For cell enumeration, at least seven fields were randomly selected, and > 1,000 cells were counted per animal.

## Results

### *Wip1*-knockout animals have reduced alveolar development during pregnancy

**Figure 1:**
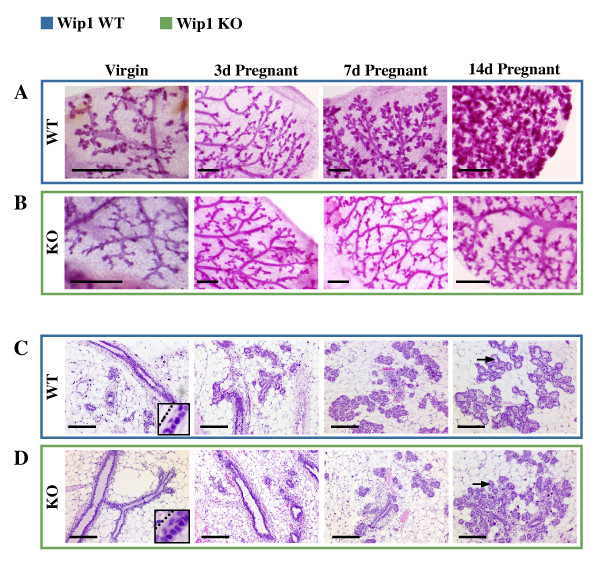
***Wip1*-knockout animals have reduced alveolar development during pregnancy**. **(A, B) **Carmine-alum-stained whole mounts of mammary glands from virgin (nulliparous) or 3-days (3d), 7-days (7d), or 14-days (14d) pregnant wild-type (WT, blue box) or *Wip1*-knockout (KO, green box) mice. Images are representative of three to five animals. Scale bar, 500 μm. **(C, D) **Hematoxylin and eosin-stained tissue sections of mammary glands from WT (blue box) or *Wip1 *KO (green box) virgin and pregnant mice, as indicated earlier. Black arrows, distention of lumens present in WT alveolar lobules at 14 days of pregnancy, which are reduced in *Wip1 *KO sections. Insets are enlarged regions to visualize bilayered epithelium. Dotted lines, location of basement membrane. Images are representative of three animals/group. Scale bar, 100 μm.

To elucidate the role of Wip1 in mammary epithelium, we assessed mammary gland development in Wip1-deficient mice at adulthood and during pregnancy. We first examined the morphology of the ductal system by carmine staining of whole mammary glands (Figure 1A, B). The mammary ducts of adult virgin females were indistinguishable between wild-type (WT) and *Wip1*-knockout (*Wip1 *KO) mice. Because the mammary gland responds to fluctuations in hormone levels across the estrus cycle by generating and regressing side branches and alveoli on a small scale, we compared each *Wip1 *KO gland with a control gland from a WT mouse in the same estrus stage (metoestrus). Examination of the ductal architecture at the cellular level with hematoxylin and eosin (H&E) staining of tissue sections (Figure 1C, D) revealed morphologically normal bilayered ducts with proper lumens in the *Wip1 *KO. To evaluate the effect of loss of Wip1 on alveolar development during pregnancy, animals were timed-mated, and glands were collected at 3, 7, and 14 days of pregnancy. In WT mammary glands, the formation of alveoli becomes evident with carmine whole-mount staining at 7 days of pregnancy, with a further increase in number and size of the alveolar lobules by day 14 of pregnancy (Figure 1A). In contrast, generation of alveolar lobules in *Wip1 *KO glands is substantially delayed. Analyses of tissue sections show that the initiation of mammary alveolar development can already be detected with H&E in 3-day pregnant WT mice, whereas this is observed only in 7-day pregnant Wip1 KO animals (Figure 1C, D). In WT mammary glands at 14 days of pregnancy, distended lumens become apparent in the developing alveoli, but in the absence of Wip1, the alveolar architecture still resembles that of the WT at 7 days of pregnancy (Figure 1C, D). It is noteworthy that *Wip1 *KO animals are eventually able to nurse their pups, indicating that alveolar development progresses all the way to functional lactation, but our analyses show an obvious delay in alveologenesis during the initial phase of pregnancy.

### Wip1 is required for STAT5 activation in a subset of luminal cells

**Figure 2:**
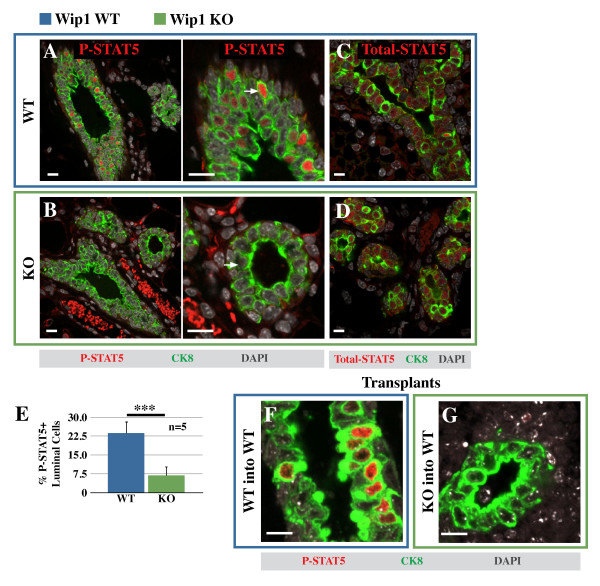
**Wip1 is required for STAT5 activation in a subset of luminal cells**. **(A, B) **Confocal immunofluorescence of mammary gland sections from virgin wild-type (WT, A) and *Wip1*-knockout (KO, B) mice, detecting phosphorylated signal transducer and activator of transcription 5 (P-STAT5, red) and the luminal cell marker cytokeratin-8 (CK8, green). Middle panels, enlarged sections of images shown in A and B. White arrows, representative luminal cells with the strongest P-STAT5-positive signal for that genotype. Red signal outside context of CK8 is nonspecific staining of erythrocytes. Control staining without primary antibody can be found in Additional file 4. **(C, D) **Same set of tissue samples probed with antibodies against STAT5 (Total-STAT5, red) and cytokeratin-8 (CK8, green). **(E) **Quantification of the percentage of phospho-STAT5-positive cells in WT (blue bar) and *Wip1 *KO mice (green bar). Values are presented as the mean proportions from five mice/group ± SD. ***P < 0.001. **(F, G) **Outgrowths from WT and *Wip1 *KO mammary epithelial cells 8 weeks after transplantation into WT cleared mammary fat pads and probed for P-STAT5 (red) and cytokeratin-8 (green). DAPI counterstain indicates cell nuclei (gray). Scale bar, 10 μm.

To determine the molecular cause of reduced alveolar development in Wip1-deficient mammary glands, we assessed the activation status of STAT5, an essential regulator of alveolar development [30]. Dual confocal immunofluorescence of phosphorylated STAT5 (the active form) and cytokeratin-8 (a marker for cells in the luminal layer) was performed on sections of fixed tissue. We first examined mammary glands from virgin animals and found strong P-STAT5 staining in a subset of luminal cells in wild-type tissue (Figure 2A, red). In contrast, P-STAT5 was very low in the absence of Wip1 (Figure 2B, with quantification in 2E). This is due to a lack of phosphorylation, because STAT5 protein expression is comparable between *Wip1 *KO and WT mammary epithelium (Figure 2C, D, red). In rare cells, weak P-STAT5 staining was detectable in *Wip1 *KO tissue (white arrow in Figure 2B), indicating that STAT5 activation was severely attenuated but not entirely abrogated. Although fluctuations in P-STAT5 were observed in WT mice across the estrus cycle, as previously reported [31], the signal for P-STAT5 remained lower in *Wip1 *KO mice compared with WT mice, independent of estrus stage (data not shown).

To exclude the possibility that the lack of STAT5 activation in *Wip1 *KO mammary epithelial cells was due a systemic defect, such as a requirement for Wip1 in prolactin production from the pituitary gland, primary mammary epithelial cells were isolated with FACS and transplanted into mammary fat pads of WT mice, from which the endogenous mammary epithelium had been removed. We found no difference in the capacity of WT or *Wip1 *KO cells to reconstitute a mammary epithelial ductal system in the cleared fat pads (data not shown). However, whereas reconstituted mammary epithelium from WT donors exhibited robust P-STAT5 immunoreactivity (Figure 2F, red), *Wip1 *KO mammary epithelial cells in the contralateral fat pad of the same animal failed to activate STAT5 (Figure 2G). This experiment demonstrates that a cell-autonomous requirement exists for Wip1 expression to activate STAT5 in mammary epithelial cells.

### Steroid-receptor-positive cells require Wip1 to respond to low levels of prolactin

**Figure 3:**
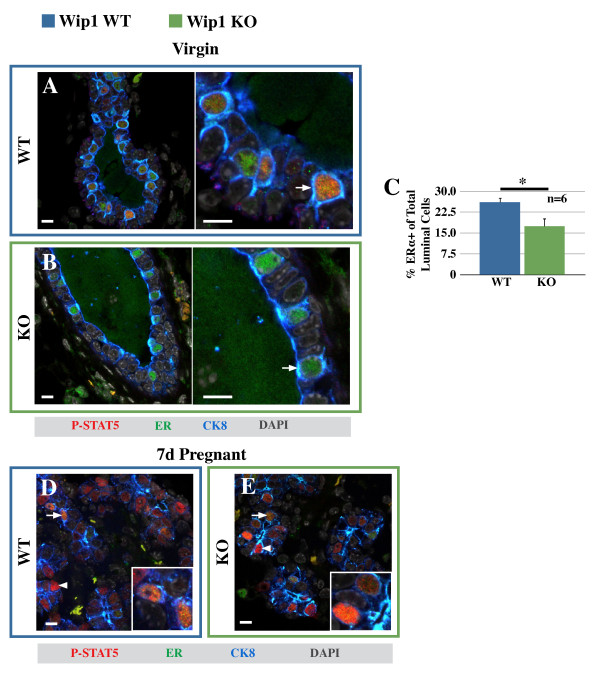
**Steroid-receptor-positive cells require Wip1 to respond to low levels of prolactin**. **(A, B) **Confocal immunofluorescence of mammary gland sections from virgin wild-type (WT, A) and *Wip1*-knockout (KO, B) mice detecting estrogen receptor-α (ER, green), phospho-STAT5 (P-STAT5, red), and cytokeratin-8 (CK8, blue). Right panels, enlarged sections of images shown in A and B. **(C) **Percentage of ER^+ ^luminal cells in virgin WT (blue bar) and *Wip1 *KO mice (green bar). Values represent the mean proportions from six mice/group ± SD. *P < 0.05. **(D, E) **Confocal immunofluorescence of 7-day pregnant WT (D) and *Wip1 *KO (E) mammary sections detecting estrogen receptor-α (ER, green), P-STAT5 (P-STAT5, red), and cytokeratin-8 (CK8, blue). Inset is enlarged section of the same image. Arrows, ER-positive cells; arrowheads, alveolar cells. Images are representative of five animals. Scale bar, 10 μm.

In wild-type mammary ducts, activated STAT5 was observed in only a subset of luminal cells. To determine whether these are alveolar cells or steroid-receptor-positive cells, co-localization of P-STAT5 with estrogen receptor-α (ER) was determined with confocal microscopy. Surprisingly, virtually all P-STAT5-positive cells were also positive for ER (Figure 3A) or the progesterone receptor (PR; see Additional file 5A), demonstrating that steroid-receptor-positive cells are the principal cells to activate STAT5 in the virgin state. Notably, Nevalainen *et al*. [32] showed that in virgin mammary epithelium, the activation of STAT5 occurs exclusively through the prolactin receptor. Steroid-receptor-positive cells have been designated "sensor cells" based on their response to estrogen and progesterone [8], but their sensitivity to prolactin further emphasizes their role as primary sensors for systemic cues, and we henceforth refer to them as hormone-sensing cells. Hormone-sensing cells stain more intensely with the cytokeratin-8 antibody (Figure 3A), and have a more cuboidal appearance compared with columnar alveolar progenitor cells [12]. The alveolar identity of the ER-negative, columnar cells is demonstrated by their expression of Elf5 ([12], Additional file 5B), and even though likely other progenitor cells occur among the ER-negative cells, for clarity purposes, ER-negative luminal cells are henceforth referred to as alveolar progenitor cells.

Thus, in WT mammary epithelium, phosphorylation of STAT5 is restricted to ER-positive cells, even though STAT5 protein is detectable in both alveolar progenitor- and hormone-sensing cells (Additional file 5C). In the absence of Wip1, STAT5 protein is still present in both cell populations (Additional file 5D), but a conspicuous absence of phosphorylated STAT5 is observed in the ER-positive cells (Figure 3B). Together, these findings raise the possibility that the hormone-sensing cells, rather than the alveolar progenitor cells, are directly affected by loss of Wip1. Accordingly, we found a small but significant reduction in the number of ER-positive cells in Wip1-deficient mammary glands (Figure 3C). In summary, these experiments indicate that Wip1 is required for hormone-sensing cells to respond to the low levels of prolactin in the virgin state. During pregnancy, prolactin levels increase 10- to 20-fold [33], and in sections from timed-mated animals at 7 days of pregnancy, P-STAT5 was observed in ER-positive and alveolar cells of both WT and *Wip1 *KO mice (Figure 3D, E). This illustrates two points: (a) defective STAT5 activation in *Wip1 *KO hormone-sensing cells is rescued in the presence of a pregnancy-associated hormonal milieu, and (b) alveolar cells appear largely unaffected by the absence of Wip1 in their response to pregnancy signals.

### Hormone-receptor expression is unaffected in the absence of Wip1

**Figure 4:**
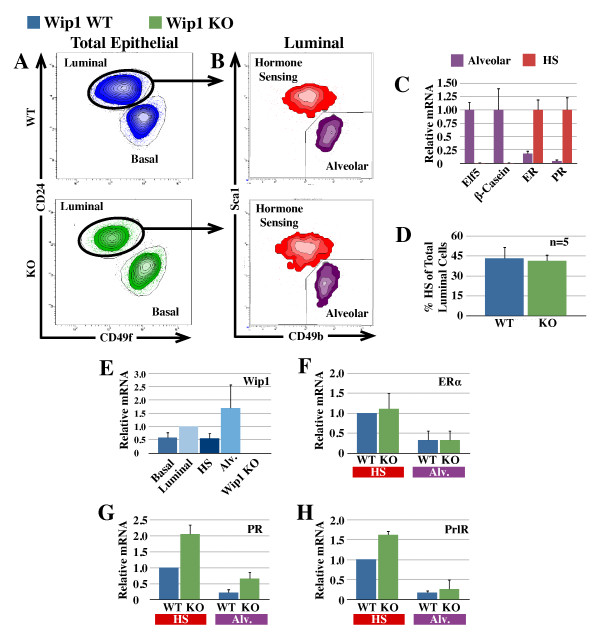
**Hormone-receptor expression is unaffected in the absence of Wip1**. **(A, B) **Flow-cytometric analysis of mammary epithelial cells isolated from virgin wild-type (WT, blue) and *Wip1*-knockout mice (KO, green) and gated to remove dead cells/debris, doublets, and lymphocytes (see gating strategy in Additional file 2). Luminal cells were separated into hormone-sensing (HS, red) and alveolar (Alv, purple) subsets based on the expression of Sca1 and CD49b (B). **(C) **Subset identity was validated with qPCR for alveolar cells (E74-like factor 5 (Elf5) and β-casein) and hormone-sensing cells (estrogen and progesterone receptor (ER and PR). **(D) **Proportion of hormone-sensing cells (Sca1^hi^CD49b^lo^) in WT (blue bars) and *Wip1 *KO mammary glands (green bars). Values are presented as the mean proportions from five mice/group ± SD. **(E) **Relative *Wip1 *mRNA proportions in individual subsets of mammary epithelial cells. **(F through H) **Relative proportions of mRNA for estrogen receptor (ERα, F), progesterone receptor (PR, G), and prolactin receptor (PrlR, H) in WT (blue bars) and *Wip1 *KO (green bars) epithelial subsets. All qPCR data (E through H) are presented as the mean ± SD for three to four individual sets of WT and *Wip1 *KO animals.

To determine whether the lack of STAT5 activation in Wip1-deficient hormone-sensing cells is due to a reduction in prolactin-receptor expression, mammary epithelial subsets were sorted for qPCR analysis. Basal and luminal subsets were identified by using CD24 and CD49f (α_6_-integrin; Figure 4A), after exclusion of debris, doublets, dead cells, and lymphocytes, as outlined in Additional file 2. This was followed by discrimination of alveolar progenitor- and hormone sensing-enriched fractions by using Sca1 (Ly6A) and CD49b (α_2_-integrin, Figure 4B). Subpopulations were validated based on the expression of alveolar (Elf5 and β-casein [34]) and hormone-sensing cell markers (ER and PR) (Figure 4C) by using a direct qPCR protocol developed for the convenient interrogation of gene expression in small numbers of cells. For each population, two to three independent tubes of 500 sorted cells were assayed per animal.

Analysis of Wip1 transcription in the cellular subsets showed that Wip1 is expressed in all mammary epithelial cells, with a higher level of transcription in alveolar progenitor cells (Figure 4E). We were unable to achieve a specific antibody staining for Wip1 protein in mouse cells, based on *Wip1 *KO control sections (data not shown), and could therefore not assess whether Wip1 protein levels reflect transcript levels. Even though *Wip1 *transcription is lower in hormone-sensing cells compared with alveolar cells, our data demonstrate a clear functional role for Wip1 in ER-positive cells (Figures 2 and 3). It is noteworthy that by FACS analysis, the proportion of hormone-sensing cells was not significantly different between WT and *Wip1 *KO mice (Figure 4D), and *ER *transcription was similar in WT and *Wip1 *KO cells (Figure 4F). This suggests that the lower proportion of ER-positive cells in *Wip1 *KO glands, when quantified by confocal immunofluorescence (Figure 3C), likely results from reduced ER protein expression/stability rather than a loss of ER-positive cells. Despite this potential reduction in ER protein, the activity of the estrogen receptor did not seem to be affected in the absence of Wip1, because *PR *transcription is dependent on estrogen [35] and *PR *transcription was not reduced in *Wip1 *KO samples (Figure 4G). Importantly, transcription of the prolactin receptor was also not reduced in Wip1-deficient cells (Figure 4H), indicating that the lack of P-STAT5 is not due to a defect in receptor expression. Together, these data highlight that receptors for steroid sex hormones and prolactin are predominantly expressed in specialized hormone-sensing cells, and their expression is not reduced in the absence of Wip1.

### Hormone-sensing cells produce less paracrine factors in the absence of Wip1

**Figure 5:**
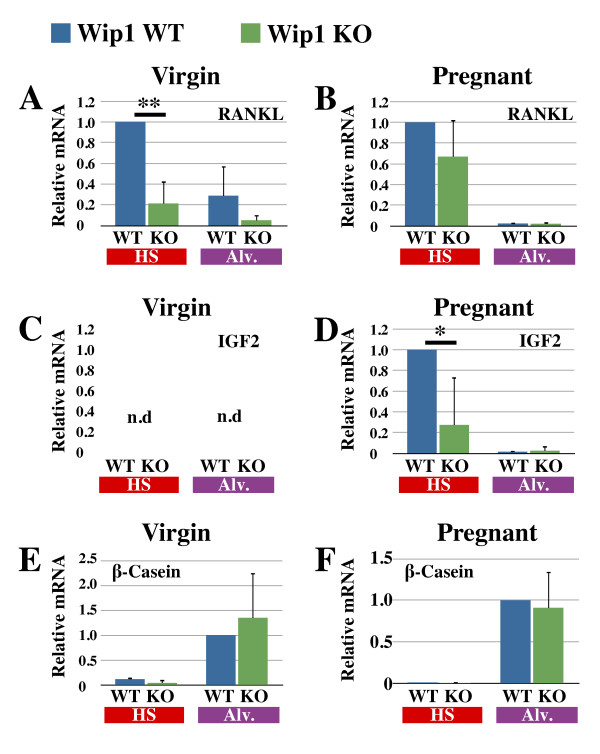
**Hormone-sensing cells produce less paracrine factors in the absence of Wip1**. Quantitative polymerase chain reaction **(**qPCR) analysis of receptor activator of nuclear factor kappa-B ligand (*RANKL*; **A, B**), insulin-like growth factor-2 (*IGF-2*; **C, D**), and β-casein **(E, F) **transcription in wild-type (WT, blue bars) and *Wip1*-knockout (KO, green bars) luminal subsets (hormone-sensing (HS, red) and alveolar cells (Alv, purple)) obtained from virgin or 7-day pregnant mice. Data are presented as mean ± SD of three to four separate sets of WT and *Wip1 *KO animals in three separate qPCR experiments. **P < 0.01, and *P < 0.05; n.d., not detectable.

Our observation that Wip1 allows hormone-sensing cells but not alveolar progenitor cells to respond to low prolactin levels raises the question: why is pregnancy-induced alveolar development delayed in *Wip1 *KO mice? To answer this question, we measured whether lack of Wip1 affected the production of paracrine factors by hormone-sensing cells, such as RANKL and IGF2. Mice deficient for either RANKL or IGF2 have defects in alveolar development in response to pregnancy [36-38]. *RANKL *is induced by progesterone and not by prolactin [38], but is absent in *Stat5*-knockout animals [39], suggesting that optimal *RANKL *transcription requires both progesterone and prolactin signaling [40,41]. Accordingly, we detected *RANKL *transcription predominantly in hormone-sensing cells (Figure 5A, B). In the absence of Wip1, a clear reduction in *RANKL *transcription was seen in virgin samples, and this reduction was still present but less pronounced in samples from 7-day pregnant animals (Figure 5A, B). *IGF2 *transcription was undetectable in virgin samples, but increased dramatically with pregnancy. It has been reported that *IGF2 *transcription is induced by prolactin [38,42], and our analysis of sorted cellular subsets from WT mammary glands demonstrated that IGF2 is produced specifically in hormone-sensing cells (Figure 5D). In *Wip1*-knockout samples, *IGF2 *transcription was significantly reduced at 7 days of pregnancy (Figure 5D), suggesting that even during pregnancy, prolactin signaling in hormone-sensing cells may not be fully active without Wip1.

Notably, transcription of the milk gene *β-casein *in an equal number of sorted alveolar cells is not reduced in the absence of Wip1 (Figure 5E, F), suggesting that prolactin signaling in alveolar cells, as detected by P-STAT5 at 7 days of pregnancy (Figure 3E), is Wip1 independent. Overall, these findings show that hormone-sensing cells produce not only RANKL but also IGF2, and limited expression of these paracrine factors in the *Wip1 *KO provides a likely explanation for the reduced alveolar development in the initial stages of pregnancy.

### Hormone-sensing cells are dependent on Wip1 for their response to HER2/neu activation

**Figure 6:**
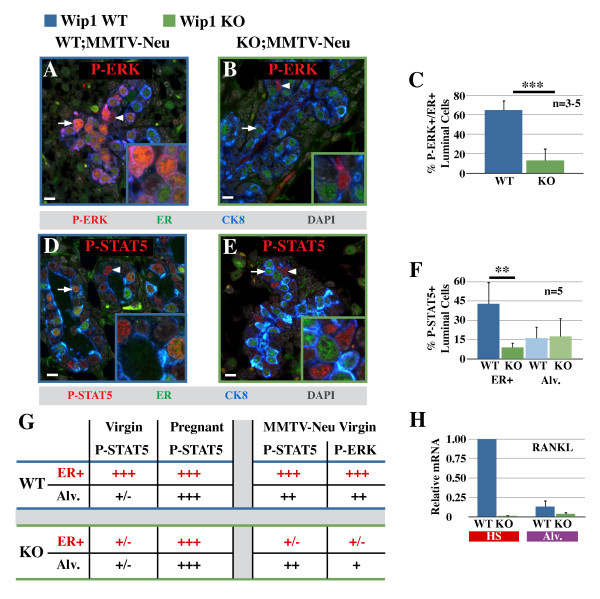
**Hormone-sensing cells are dependent on Wip1 for their response to Her2/neu activation**. **(A, B) **Confocal immunofluorescence detecting phosphorylated extracellular signal-regulated kinase (P-ERK, red), estrogen receptor-α (ER, green), and cytokeratin-8 (blue) in MMTV (mouse mammary tumor virus)-*neu Wip1 *wild-type (WT; MMTV-*neu*, A) or MMTV-*neu Wip1*-knockout (KO; MMTV-*neu*, B) mammary epithelium. **(C) **Quantification of the proportion of ER-positive (ER^+^) luminal cells positive for phospho-ERK. Values are presented as the mean proportions from three to five mice/group ± SD. **P < 0.001. (D, E) Confocal immunofluorescence detecting phosphorylated signal transducer and activator of transcription 5 (P-STAT5, red), estrogen receptor-α (ER, green), and cytokeratin-8 (CK8, blue) in MMTV-*neu Wip1 *wild-type (WT; MMTV-*neu*, D) or MMTV-*neu Wip1 *KO (KO; MMTV-*neu*, E) mammary epithelium. Scale bar, 10 μm. Arrows, ER-positive cells; arrowheads, alveolar cells. **(F) **Quantification of the proportion of ER-positive or ER-negative luminal cells positive for phospho-STAT5. Values are presented as the mean proportions from five mice/group ± SD. ***P < 0.01. **(G) **Summary of immunostaining intensities for phospho-STAT5 and phospho-ERK in Neu-positive wild-type and *Wip1 *KO virgin and pregnant mice, and in the context of MMTV-*neu*. **(H) **Receptor activator of nuclear factor kappa-B ligand (RANKL) transcription in Neu-positive wild-type (blue bars) and *Wip1 *KO (green bars) luminal subsets (hormone sensing (HS, red) and alveolar cells (purple)). Data are presented as mean ± SD of two separate sets of wild-type and *Wip1 *KO animals.

Thus far we have identified a surprising role for Wip1 in the function of hormone-sensing cells rather than of alveolar progenitor cells, and this prompted us to investigate how these different cell types respond to HER2/neu activation in the presence or absence of Wip1. To this end, MMTV-*neu *mice were crossed with *Wip1 *KO mice, and mammary glands from MMTV-*neu*;*Wip1 *WT and MMTV-*neu*;*Wip1 *KO mice were fixed, sectioned, and immunostained for phosphorylated ERK (P-ERK) and P-STAT5. Interestingly, phosphorylation of ERK by HER2/neu activation was more pronounced in hormone-sensing cells compared with alveolar progenitor cells (Figure 6A). In the absence of Wip1, ERK activation by HER2/neu in hormone-sensing cells was significantly reduced (Figure 6B, quantified in 6C).

In mammary glands expressing *Wip1*, P-STAT5 was detectable in hormone-sensing cells, as before (Figures 3A, 6D and 6G). We did not detect P-STAT5 in alveolar progenitor cells in virgin mammary glands (Figure 3A), but strikingly, in the presence of activated HER2/neu, STAT5 was phosphorylated in alveolar progenitor cells as well (Figure 6D). Likewise, in mammary glands from virgin *Wip1*-knockout animals, alveolar progenitor cells are positive for P-STAT5 in the presence of activated HER2/neu (Figure 6E), demonstrating that this effect is Wip1 independent. In contrast, the proportion of P-STAT5-positive hormone-sensing cells was still significantly reduced in the absence of Wip1 (Figure 6E). Thus, the defect in STAT5 activation in *Wip1 *KO hormone-sensing cells persists in the presence of activated HER2/neu, but both wild-type and *Wip1 *KO alveolar progenitor cells respond to HER2/neu by activating STAT5 (Figure 6F). These findings demonstrate that HER2/neu signaling is active in Wip1-deficient alveolar progenitor cells, the presumptive cells of origin for MMTV-*neu *tumorigenesis. In contrast, hormone-sensing cells require Wip1 to respond to HER2/neu activation with either ERK or STAT5 activation (Figure 6G), highlighting the importance of cell context in signal transduction. qPCR data on cell subsets sorted from MMTV-*neu *mammary glands demonstrated that *RANKL *transcription in hormone-sensing cells remains low in the absence of Wip1, even when HER2/neu is activated (Figure 6H), consistent with the lack of STAT5 activation in these cells. Interestingly, hormone-sensing cells are intermingled with ER-negative cells in intraductal lesions of MMTV-*neu *mammary glands (see Additional file 6), raising the possibility that paracrine stimulation and Wip1 activity continue to play a role at this later stage of tumorigenesis.

## Discussion

### Wip1 potentiates the response of hormone-sensing cells to prolactin

In adult mammary glands of virgin mice, we found that Wip1 is required for STAT5 activation, specifically in hormone-sensing cells. Because of the obvious requirement for prolactin signaling and STAT5 activation in alveolar development and milk production, the role of STAT5 in alveolar cells has received the most attention [19,43]. We showed for the first time that phosphorylated STAT5 colocalizes only with ER- and PR-positive cells in mammary epithelium of nonmanipulated virgin animals. Because phosphorylation of STAT5 in virgin mammary epithelium is strictly dependent on the presence of the prolactin receptor [32], our data demonstrate that hormone-sensing cells are the principal responders to prolactin in the virgin state. This is consistent with previous studies that described a similar pattern for progesterone-receptor and prolactin-receptor expression in virgin mammary glands [44,45]. Moreover, a study with ovariectomized mice showed that soon after estrogen and progesterone injection, STAT5 was localized to the nucleus of steroid-receptor-positive cells specifically, with translocation to the cytoplasm on inhibition of pituitary prolactin secretion [46], again illustrating the capacity of hormone-sensing cells to respond to prolactin.

During pregnancy, when prolactin levels increase substantially [33], we observed phosphorylated STAT5 not only in the hormone-sensing cells, but also in alveolar cells. Others have shown that injection of supraphysiologic levels of prolactin caused STAT5 activation in all luminal cells, in contrast to the scattered pattern observed in the nonmanipulated state [17,32]. This strongly suggests that the higher levels of prolactin during pregnancy activate STAT5 in alveolar cells, rather than alternative pregnancy-induced signaling pathways. Altogether, these findings indicate that although alveolar cells are capable of responding directly to prolactin, their threshold for STAT5 activation is considerably higher than that of hormone-sensing cells.

Strikingly, the ability of hormone-sensing cells to respond to low levels of prolactin is strictly dependent on Wip1 expression, as indicated by virtually undetectable levels of activated STAT5 in *Wip1*-knockout mammary epithelium. STAT5 activation in Wip1-deficient hormone-sensing cells is rescued by day 7 of pregnancy, suggesting that hormone-sensing cells are able to activate STAT5 in the absence of Wip1 when prolactin levels are high enough, but require Wip1 to potentiate the signal transduction in the virgin state. Even though *Wip1 *is expressed in alveolar progenitor cells, activated STAT5 is not detectable in the virgin state, which implies that the target for Wip1 that allows potentiation of prolactin signaling is either not present or not available in alveolar progenitor cells. It is currently unclear what the relevant target is for Wip1 in hormone-sensing cells that allows STAT5 activation. Several targets for Wip1 have been identified, including various proteins involved in DNA-damage signaling, as well as the stress kinase p38MAPK [6]. Although we cannot rule out at this stage that prolonged DNA-damage signaling and p53 activation prevent STAT5 activation, hyperactivation of p38MAPK in the absence of Wip1 seems a more likely cause of the lack of P-STAT5, based on the observation that p38MAPK inhibits JAK-STAT signaling in monocytes [47] and because treatment of MMTV-*neu*;*Wip1 *KO animals with a p38MAPK inhibitor restored tumorigenesis, at least partially [4]. Unfortunately, the increased sensitivity of hormone-sensing cells to prolactin is lost when primary mammary epithelial cells are taken into culture (data not shown), further emphasizing the importance of cell and tissue context for the role of Wip1 in mammary tumorigenesis and highlighting the need for more sophisticated mouse models to dissect the molecular mechanism.

### Different role for prolactin signaling in hormone-sensing versus alveolar cells

Our data show that cell context is also important for the downstream effect of prolactin-receptor activation. For instance, STAT5 activation results in milk-gene transcription only in alveolar cells and not in hormone-sensing cells. Experiments in cell lines suggest that both ER and PR can prevent binding of STAT5 to the *β-casein *promoter [48,49], illustrating how the molecular circuitry of a particular cell type can direct the transcriptional response to, for example, prolactin signaling. Similarly, we showed that *IGF2 *transcription occurs in hormone-sensing cells but not alveolar cells when both cells are responding to prolactin (at 7 days of pregnancy). Whether *IGF2 *is a direct target for STAT5 in hormone-sensing cells [38,50] and how its transcription is prevented in alveolar cells remains to be established. Interestingly, the *IGF2*-knockout mouse phenocopies the defect in alveologenesis observed in the Wip1-knockout mouse. In both cases, a considerable delay in alveolar development occurs during the first half of pregnancy, and this is rescued late in pregnancy, and *IGF2 *KO as well as *Wip1 *KO animals are capable of nursing their pups ([38] and DB/AP unpublished observation). Ectopic *IGF2 *expression rescues alveolar morphogenesis but not milk-gene transcription in prolactin-receptor knockout mammary epithelium [38]. Together with our data, this suggests that the initial phase of alveologenesis is dependent on prolactin signaling relayed by hormone-sensing cells, whereas prolactin signaling in alveolar cells themselves is required during the later stages of pregnancy to initiate milk production.

Hormone-sensing cells also transcribe less *RANKL *in the absence of Wip1. It has been shown that *RANKL *expression is dependent on progesterone [51]; however, it is currently unknown whether PR activity is reduced in Wip1 KO mice. In luciferase promoter assays using cancer cells, Wip1 was shown to enhance both ER and PR activity [52], but we do not observe a decrease in *PR *transcription, suggesting that ER activity is not affected by Wip1 loss. Considering that *RANKL *expression is substantially reduced in *Stat5*-knockout mice [39], we interpret the lack of *IGF2 *and *RANKL *expression by *Wip1 *KO hormone-sensing cells to be due to reduced prolactin signaling. Both paracrine factors have been shown to be important for promoting alveolar development [38,53], providing an explanation for the reduced alveologenesis in *Wip1*-knockout animals.

### The role of hormone-sensing cells in early tumorigenesis

We found a defect in STAT5 activation in Wip1-deficient hormone-sensing cells, even in the presence of activated HER2/neu. Several studies demonstrate that interfering with hormone-sensing cell function delays mammary tumorigenesis. For instance, tamoxifen treatment of young MMTV-*neu *mice results in a delay in tumor formation that is uncannily similar to the one observed in the absence of Wip1 [4,54]. Interestingly, tamoxifen not only inhibits estrogen signaling, but it also reduces serum prolactin levels [55] and prevents prolactin binding to its receptor [56], raising the possibility that a reduction in STAT5 activity was responsible for reduced tumor formation in this setting. Notably, once the (ER-negative) tumors had developed, tamoxifen treatment did not inhibit their growth [54], highlighting the specific requirement for functional hormone-sensing cells during premalignant development. Tamoxifen treatment also delayed tumorigenesis in other mouse models of estrogen-receptor-negative mammary tumors [55], and the lack of prolactin-receptor expression reduced proliferation in early lesions and delayed SV40-driven tumorigenesis, but did not affect growth of the tumors once they occurred [57]. Similarly, deletion of *Jak2 *from mammary epithelial cells in general protected against tumor development in the MMTV-*neu *model, but deletion of *Jak2 *from tumor cells did not affect their proliferation [58]. Finally, pharmacologic inhibition of RANKL strongly reduced the number of premalignant lesions in MMTV-*neu *mice [59]. Thus, the absence of active STAT5 in *Wip1 *KO hormone-sensing cells and the subsequent paucity of RANKL may be sufficient to explain a delay in tumorigenesis.

Although alveolar progenitors are thought to be the cells of origin for tumors in the MMTV-*neu *model, we showed for the first time that HER2/neu activation triggers a response in hormone-sensing cells, as indicated by ERK activation, and this response is severely attenuated in the absence of Wip1. Clearly, the MMTV-*neu *model is different from sporadic tumorigenesis in that the MMTV LTR drives activated *HER2/neu *expression in multiple cell types simultaneously, including both hormone-sensing and alveolar progenitor cells [28,60]. In a different mouse model, activated *HER2/neu *is expressed by the endogenous promoter; mimicking human HER2^+ ^breast cancer more closely. Even though the tumors that arise in this model also express milk genes [61], it is presently unclear what the target cell is for transformation by HER2 in the human breast. At least a subset of HER2^+ ^breast cancers are ER^+ ^[62], raising the possibility that these tumors arise from transformation of cells in the hormone-sensing lineage. It will be important to find out whether human steroid-receptor-positive cells also require Wip1 for their response to prolactin and HER2/neu activation. This is particularly relevant because women with elevated serum prolactin levels have an increased risk of breast cancer [63]. Our findings highlight that prolactin signaling in hormone-sensing cells contributes to the growth-promoting rather than to the differentiation-inducing effects of prolactin. It seems that alveolar progenitor cells are especially dependent on this paracrine stimulation in early pregnancy and at the early stages of tumorigenesis. Thus, inhibiting the function of hormone-sensing cells might reduce the occurrence not only of ER^+ ^breast cancer, but could also hamper premalignant development of ER^- ^breast cancer [54,64,65]. Currently, Wip1 inhibitors are under development [66], prompted by the observation that cells from established tumors with *Wip1 *amplification remain dependent on Wip1 for their survival [67]. Although our study does not address the effect of *Wip1 *over-expression in tumor cells, our data do suggest that it would be worthwhile to explore the use of Wip1 inhibitors for preventive treatment, similar to the recently approved use of tamoxifen in women with a high risk of breast cancer [55,68]. Also, the addition of Wip1 inhibitors as adjuvant therapy to standard chemotherapeutic regimens may be of use in extending recurrence-free survival.

Overall, our study underscores the relevance of cell context in signal transduction and highlights the role of hormone-sensing cells as integrators of systemic signals and their subsequent influence on normal and premalignant development.

## Conclusions

**Figure 7:**
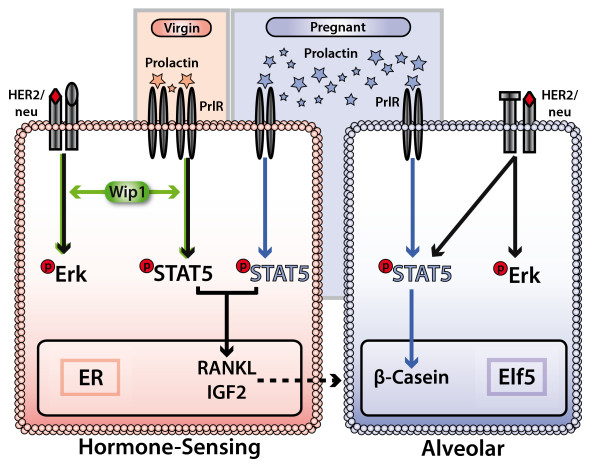
**Model for the cell-type-specific role of Wip1 in the mammary gland**. In the virgin state, Wip1 (also known as PPM1D; protein phosphatase magnesium-dependent 1D) is required to sensitize hormone-sensing cells (HS, red) to prolactin by promoting phosphorylation of signal transducer and activator of transcription 5 (STAT5), whereas STAT5 activation is undetectable in adjacent alveolar progenitor cells (Alv, purple), even when Wip1 is expressed. During pregnancy, prolactin levels increase, and STAT5 is activated in both hormone-sensing and alveolar progenitor cells (blue arrows), independent of Wip1. In the absence of Wip1, STAT5-induced transcription of β-casein in alveolar cells is unaffected, but in hormone-sensing cells, transcription of paracrine regulators *RANKL *(receptor activator of nuclear factor kappa-B ligand) and *IGF2 *(insulin-like growth factor-2) is significantly reduced. In the context of HER2/neu (human epidermal growth factor receptor 2) activation, STAT5 is phosphorylated in alveolar progenitor cells independent of Wip1, but Wip1 is required for both STAT5 and ERK (extracellular signal-regulated kinase) activation in hormone-sensing cells. Thus, Wip1 potentiates prolactin and HER2/neu signaling specifically in hormone-sensing cells and is important for the production of paracrine stimulators of alveolar development.

We showed that distinct mammary epithelial cell types respond differently to prolactin signaling (Figure 7). Specifically, hormone-receptor-positive cells already activate STAT5 in the virgin state and transcribe the paracrine factors *RANKL *and *IGF2*. In contrast, alveolar progenitor cells detect prolactin only during pregnancy where and STAT5 activation results in milk-gene transcription. The Wip1 phosphatase potentiates prolactin signaling and is required for ERK activation by HER2/neu in hormone-sensing cells but not in alveolar progenitor cells. Therefore, the delay in MMTV-*neu *tumorigenesis in the absence of Wip1 is likely due to a lack of paracrine stimulation of alveolar progenitor cells. Overall, our findings underscore the relevance of cell context in signal transduction and suggest a novel strategy to prevent breast cancer progression: indirectly, by inhibiting the hormone-sensing cells in their role as central conductors of proliferation.

## Abbreviations

ATM: ataxia telangiectasia mutated; Chk2: checkpoint kinase 2; DAPI: 4',6-diamidino-2-phenylindole; DMEM: Dulbecco modified Eagle medium; EDTA: ethylenediaminetetraacetic acid; Elf5: E74-like factor 5; ER: estrogen receptor; ERK: extracellular signal-regulated kinase; FCS: fetal calf serum; FSC: forward scatter; H&E: hematoxylin and eosin; HER2: human epidermal growth factor receptor 2, also known as neu or ErbB2; HPRT: hypoxanthine-guanine phosphoribosyltransferase; IGF2: insulin-like growth factor 2; KO: knockout; MAPK: mitogen-activated protein kinase; MMTV: mouse mammary tumor virus; MMTV-*neu*: mouse mammary tumor virus promoter driving the activated form of rat *Erbb2 *oncogene; P-: the phosphorylated form of the protein before which it appears in text; PBS: phosphate-buffered saline; PI-MECs: parity-identified mammary epithelial cells; PP2C: protein phosphatase 2C; PPM1D: protein phosphatase magnesium-dependent 1D, gene name for Wip1; PR: progesterone receptor; qPCR: quantitative polymerase chain reaction; RANKL: receptor activator of nuclear factor kappa-B ligand, also known as TNFSF11; SSC: side scatter; STAT5: signal transducer and activator of transcription; WAP: whey-acidic protein; Wip1: wild-type p53-induced phosphatase 1, also known as PPM1D; WT: wild type.

## Competing interests

The authors declare that they do not have competing interests.

## Authors' contributions

GT and DS carried out the mouse studies, including tissue and molecular analyses. DS, VH, and KK analyzed and sorted primary cells with FACS for qPCR analyses and participated in the design of the experiments. KG and BT took care of the mice and performed the genotyping. DB and AP conceived of the study. GT and AP designed and coordinated the study and drafted the manuscript. All authors read and approved the final manuscript.

## Supplementary Material

Additional file 1 **Specifications for antibodies used in confocal immunofluorescence and fluorescence-activated cell sorting (FACS) analysis**. Click here for file

Additional file 2 **Gating strategy used in all FACS analysis and sorting experiments**. Click here for file

Additional file 3 **Nucleic acid sequences for primers used in quantitative polymerase chain reaction (qPCR) experiments**. Click here for file

Additional file 4 **Images of confocal immunofluorescence controls: sections of mammary tissue probed with goat-anti-mouse Alexa 488, goat-anti-rabbit Alexa 568 (A), and donkey anti-rat Alexa 633 (A, B) without the addition of primary antibody**. Click here for file

Additional file 5 **Confocal immunofluorescence of mammary tissue section probed for progesterone receptor, total STAT5 and Elf5**. Confocal immunofluorescence of mammary tissue from virgin wild-type mice probed for progesterone receptor & phosphorylated STAT5 (A) and Elf5 & cytokeratin 8 (B). Confocal immunofluorescence of mammary tissue from virgin wild-type (blue box) and *Wip1 *KO (green box) mice probed for total STAT5 and estrogen receptor (C, D). Click here for file

Additional file 6 **Confocal immunofluorescence of virgin mouse mammary tumor virus (MMTV)-*neu *mammary tissue sections probed with antibodies specific for HER2/neu, estrogen receptor, and cytokeratin-8**. Click here for file

## References

[B1] Ince TA Richardson AL Bell GW Saitoh M Godar S Karnoub AE Iglehart JD Weinberg RA Transformation of different human breast epithelial cell types leads to distinct tumor phenotypes Cancer Cell 2007 12 160 170 10.1016/j.ccr.2007.06.013 17692807

[B2] Visvader JE Cells of origin in cancer Nature 2011 469 314 322 10.1038/nature09781 21248838

[B3] Garraway LA Sellers WR Lineage dependency and lineage-survival oncogenes in human cancer Nat Rev Cancer 2006 6 593 602 10.1038/nrc1947 16862190

[B4] Bulavin DV Phillips C Nannenga B Timofeev O Donehower LA Anderson CW Appella E Fornace AJ Inactivation of the Wip1 phosphatase inhibits mammary tumorigenesis through p38 MAPK-mediated activation of the p16(Ink4a)-p19(Arf) pathway Nat Genet 2004 36 343 350 10.1038/ng1317 14991053

[B5] Lambros MB Natrajan R Geyer FC Lopez-Garcia MA Dedes KJ Savage K Lacroix-Triki M Jones RL Lord CJ Linardopoulos S Ashworth A Reis-Filho JS PPM1D gene amplification and overexpression in breast cancer: a qRT-PCR and chromogenic in situ hybridization study Mod Pathol 2010 23 1334 1345 10.1038/modpathol.2010.121 20543821

[B6] Zhu Y-H Bulavin DV Wip1-dependent signaling pathways in health and diseases Prog Mol Biol Transl Sci 2012 106 307 325 22340722 10.1016/B978-0-12-396456-4.00001-8

[B7] Demidov ON Kek C Shreeram S Timofeev O Fornace AJ Appella E Bulavin DV The role of the MKK6/p38 MAPK pathway in Wip1-dependent regulation of ErbB2-driven mammary gland tumorigenesis Oncogene 2007 26 2502 2506 10.1038/sj.onc.1210032 17016428

[B8] Brisken C Duss S Stem cells and the stem cell niche in the breast: an integrated hormonal and developmental perspective Stem Cell Rev 2007 3 147 156 10.1007/s12015-007-0019-1 17873347

[B9] Clarke RB Howell A Potten CS Anderson E Dissociation between steroid receptor expression and cell proliferation in the human breast Cancer Res 1997 57 4987 4991 9371488

[B10] Brisken C O'Malley B Hormone action in the mammary gland Cold Spring Harbor Perspect Biol 2010 2 a003178 a003178 10.1101/cshperspect.a003178 PMC2982168 20739412

[B11] Sleeman KE Kendrick H Robertson D Isacke CM Ashworth A Smalley MJ Dissociation of estrogen receptor expression and in vivo stem cell activity in the mammary gland J Cell Biol 2007 176 19 26 10.1083/jcb.200604065 17190790 PMC2063618

[B12] Harris J Stanford PM Sutherland K Oakes SR Naylor MJ Robertson FG Blazek KD Kazlauskas M Hilton HN Wittlin S Alexander WS Lindeman GJ Visvader JE Ormandy CJ Socs2 and elf5 mediate prolactin-induced mammary gland development Mol Endocrinol 2006 20 1177 1187 10.1210/me.2005-0473 16469767

[B13] Visvader JE Keeping abreast of the mammary epithelial hierarchy and breast tumorigenesis Genes Dev 2009 23 2563 2577 10.1101/gad.1849509 19933147 PMC2779757

[B14] Oakes SR Hilton HN Ormandy CJ The alveolar switch: coordinating the proliferative cues and cell fate decisions that drive the formation of lobuloalveoli from ductal epithelium Breast Cancer Res 2006 8 207 10.1186/bcr1411 16677418 PMC1557712

[B15] Richert MM Schwertfeger KL Ryder JW Anderson SM An atlas of mouse mammary gland development J Mammary Gland Biol Neoplasia 2000 5 227 241 10.1023/A:1026499523505 11149575

[B16] Ormandy CJ Binart N Kelly PA Mammary gland development in prolactin receptor knockout mice J Mammary Gland Biol Neoplasia 1997 2 355 364 10.1023/A:1026395229025 10935023

[B17] Wagner K-U Krempler A Triplett AA Qi Y George NM Zhu J Rui H Impaired alveologenesis and maintenance of secretory mammary epithelial cells in Jak2 conditional knockout mice Mol Cell Biol 2004 24 5510 5520 10.1128/MCB.24.12.5510-5520.2004 15169911 PMC419899

[B18] Liu XX Robinson GWG Wagner KUK Garrett LL Wynshaw-Boris AA Hennighausen LL Stat5a is mandatory for adult mammary gland development and lactogenesis Genes Dev 1997 11 179 186 10.1101/gad.11.2.179 9009201

[B19] Reichenstein M Rauner G Barash I Conditional repression of STAT5 expression during lactation reveals its exclusive roles in mammary gland morphology, milk-protein gene expression, and neonate growth Mol Reprod Dev 2011 78 585 596 10.1002/mrd.21345 21688337

[B20] Wagner K-U Rui H Jak2/Stat5 signaling in mammogenesis, breast cancer initiation and progression J Mammary Gland Biol Neoplasia 2008 13 93 103 10.1007/s10911-008-9062-z 18228120

[B21] Hughes K Watson CJ The spectrum of STAT functions in mammary gland development Jak-stat 2012 1 151 158 10.4161/jkst.19691 24058764 PMC3670238

[B22] Li Y Welm B Podsypanina K Huang S Chamorro M Zhang X Rowlands T Egeblad M Cowin P Werb Z Tan L Rosen J Varmus H Evidence that transgenes encoding components of the Wnt signaling pathway preferentially induce mammary cancers from progenitor cells Proc Natl Acad Sci USA 2003 100 15853 15858 10.1073/pnas.2136825100 14668450 PMC307657

[B23] Zeng YA Nusse R Wnt proteins are self-renewal factors for mammary stem cells and promote their long-term expansion in culture Stem Cell 2010 6 568 577 10.1016/j.stem.2010.03.020 PMC2917779 20569694

[B24] Henry MD Triplett AA Oh KB Smith GH Wagner K-U Parity-induced mammary epithelial cells facilitate tumorigenesis in MMTV-neu transgenic mice Oncogene 2004 23 6980 6985 10.1038/sj.onc.1207827 15286714

[B25] Boulanger CA Wagner K-U Smith GH Parity-induced mouse mammary epithelial cells are pluripotent, self-renewing and sensitive to TGF-beta1 expression Oncogene 2005 24 552 560 10.1038/sj.onc.1208185 15580303

[B26] Jeselsohn R Brown NE Arendt L Klebba I Hu MG Kuperwasser C Hinds PW Cyclin D1 kinase activity is required for the self-renewal of mammary stem and progenitor cells that are targets of MMTV-ErbB2 tumorigenesis Cancer Cell 2010 17 65 76 10.1016/j.ccr.2009.11.024 20129248 PMC2818730

[B27] Choi J Nannenga B Demidov ON Bulavin DV Cooney A Brayton C Zhang Y Mbawuike IN Bradley A Appella E Donehower LA Mice deficient for the wild-type p53-induced phosphatase gene (Wip1) exhibit defects in reproductive organs, immune function, and cell cycle control Mol Cell Biol 2002 22 1094 1105 10.1128/MCB.22.4.1094-1105.2002 11809801 PMC134641

[B28] Muller WJ Sinn E Pattengale PK Wallace R Leder P Single-step induction of mammary adenocarcinoma in transgenic mice bearing the activated c-neu oncogene Cell 1988 54 105 115 2898299 10.1016/0092-8674(88)90184-5

[B29] Smalley MJ Isolation, culture and analysis of mouse mammary epithelial cells Methods Mol Biol 2010 633 139 170 10.1007/978-1-59745-019-5_11 20204626

[B30] Miyoshi K Shillingford JM Smith GH Grimm SL Wagner KU Oka T Rosen JM Robinson GW Hennighausen L Signal transducer and activator of transcription (Stat) 5 controls the proliferation and differentiation of mammary alveolar epithelium J Cell Biol 2001 155 531 542 10.1083/jcb.200107065 11706048 PMC2198867

[B31] Liu X Robinson GW Hennighausen L Activation of Stat5a and Stat5b by tyrosine phosphorylation is tightly linked to mammary gland differentiation Mol Endocrinol 1996 10 1496 1506 10.1210/me.10.12.1496 8961260

[B32] Nevalainen MT Xie J Bubendorf L Wagner K-U Rui H Basal activation of transcription factor signal transducer and activator of transcription (Stat5) in nonpregnant mouse and human breast epithelium Mol Endocrinol 2002 16 1108 1124 10.1210/me.16.5.1108 11981045

[B33] Larsen CM Grattan DR Prolactin-induced mitogenesis in the subventricular zone of the maternal brain during early pregnancy is essential for normal postpartum behavioral responses in the mother Endocrinology 2010 151 3805 3814 10.1210/en.2009-1385 20484459

[B34] Oakes SR Naylor MJ Asselin-Labat M-L Blazek KD Gardiner-Garden M Hilton HN Kazlauskas M Pritchard MA Chodosh LA Pfeffer PL Lindeman GJ Visvader JE Ormandy CJ The Ets transcription factor Elf5 specifies mammary alveolar cell fate Genes Dev 2008 22 581 586 10.1101/gad.1614608 18316476 PMC2259028

[B35] Haslam SZ Shyamala G Effect of oestradiol on progesterone receptors in normal mammary glands and its relationship with lactation Biochem J 1979 182 127 131 496902 10.1042/bj1820127 PMC1161241

[B36] Fata JE Kong YY Li J Sasaki T Irie-Sasaki J Moorehead RA Elliott R Scully S Voura EB Lacey DL Boyle WJ Khokha R Penninger JM The osteoclast differentiation factor osteoprotegerin-ligand is essential for mammary gland development Cell 2000 103 41 50 10.1016/S0092-8674(00)00103-3 11051546

[B37] Beleut M Rajaram RD Caikovski M Ayyanan A Germano D Choi Y Schneider P Brisken C Two distinct mechanisms underlie progesterone-induced proliferation in the mammary gland Proc Natl Acad Sci USA 2010 107 2989 2994 10.1073/pnas.0915148107 20133621 PMC2840294

[B38] Brisken C Ayyannan A Nguyen C Heineman A Reinhardt F Tan J Dey SK Dotto GP Weinberg RA Jan T IGF-2 is a mediator of prolactin-induced morphogenesis in the breast Dev Cell 2002 3 877 887 10.1016/S1534-5807(02)00365-9 12479812

[B39] Santos SJ Haslam SZ Conrad SE Signal transducer and activator of transcription 5a mediates mammary ductal branching and proliferation in the nulliparous mouse Endocrinology 2010 151 2876 2876 10.1210/en.2009-1282 20392833 PMC2875824

[B40] Srivastava S Matsuda M Hou Z Bailey JP Kitazawa R Herbst MP Horseman ND Receptor activator of NF-kappaB ligand induction via Jak2 and Stat5a in mammary epithelial cells J Biol Chem 2003 278 46171 46178 10.1074/jbc.M308545200 12952963

[B41] Lee HJ Ormandy CJ Interplay between progesterone and prolactin in mammary development and implications for breast cancer Mol Cell Endocrinol 2012 357 101 107 10.1016/j.mce.2011.09.020 21945475

[B42] Hovey RCR Harris JJ Hadsell DLD Lee AVA Ormandy CJC Vonderhaar BKB Local insulin-like growth factor-II mediates prolactin-induced mammary gland development Mol Endocrinol 2003 17 460 471 10.1210/me.2002-0214 12554791

[B43] Furth PA Nakles RE Millman S Díaz-Cruz ES Cabrera MC Signal transducer and activator of transcription 5 as a key signaling pathway in normal mammary gland developmental biology and breast cancer Breast Cancer Res 2011 13 220 220 10.1186/bcr2921 22018398 PMC3262193

[B44] Hovey RCR Trott JFJ Ginsburg EE Goldhar AA Sasaki MMM Fountain SJS Sundararajan KK Vonderhaar BKB Transcriptional and spatiotemporal regulation of prolactin receptor mRNA and cooperativity with progesterone receptor function during ductal branch growth in the mammary gland Dev Dyn 2001 222 192 205 10.1002/dvdy.1179 11668597

[B45] Grimm SL Seagroves TN Kabotyanski EB Hovey RC Vonderhaar BK Lydon JP Miyoshi K Hennighausen L Ormandy CJ Lee AV Stull MA Wood TL Rosen JM Disruption of steroid and prolactin receptor patterning in the mammary gland correlates with a block in lobuloalveolar development Mol Endocrinol 2002 16 2675 2691 10.1210/me.2002-0239 12456789

[B46] Santos SJ Haslam SZ Conrad SE Estrogen and progesterone are critical regulators of Stat5a expression in the mouse mammary gland Endocrinology 2007 149 329 338 10.1210/en.2007-0594 17884938 PMC2194608

[B47] Ahmed ST Mayer A Ji J-D Ivashkiv LB Inhibition of IL-6 signaling by a p38-dependent pathway occurs in the absence of new protein synthesis J Leukoc Biol 2002 72 154 162 12101275

[B48] Faulds MHM Pettersson KK Gustafsson JAJ Haldosén LAL Cross-talk between ERs and signal transducer and activator of transcription 5 is E2 dependent and involves two functionally separate mechanisms Mol Endocrinol 2001 15 1929 1940 10.1210/me.15.11.1929 11682624

[B49] Buser AC Obr AE Kabotyanski EB Grimm SL Rosen JM Edwards DP Progesterone receptor directly inhibits β-casein gene transcription in mammary epithelial cells through promoting promoter and enhancer repressive chromatin modifications Mol Endocrinol 2011 25 955 968 10.1210/me.2011-0064 21527503 PMC3386529

[B50] Viengchareun S Servel N Fève B Freemark M Lombès M Binart N Prolactin receptor signaling is essential for perinatal brown adipocyte function: a role for insulin-like growth factor-2 PLoS ONE 2008 3 e1535 10.1371/journal.pone.0001535 18253483 PMC2212135

[B51] Mulac-Jericevic B Lydon JP DeMayo FJ Conneely OM Defective mammary gland morphogenesis in mice lacking the progesterone receptor B isoform Proc Natl Acad Sci USA 2003 100 9744 9749 10.1073/pnas.1732707100 12897242 PMC187836

[B52] Proia DA Nannenga BW Donehower LA Weigel NL Dual roles for the phosphatase PPM1D in regulating progesterone receptor function J Biol Chem 2006 281 7089 7101 10.1074/jbc.M511839200 16352595

[B53] Mukherjee A Soyal SM Li J Ying Y He B DeMayo FJ Lydon JP Targeting RANKL to a specific subset of murine mammary epithelial cells induces ordered branching morphogenesis and alveologenesis in the absence of progesterone receptor expression FASEB J 2010 24 4408 4419 10.1096/fj.10-157982 20605949 PMC2974417

[B54] Ménard S Aiello P Tagliabue E Rumio C Lollini PL Colnaghi MI Balsari A Tamoxifen chemoprevention of a hormone-independent tumor in the proto-neu transgenic mice model Cancer Res 2000 60 273 275 10667575

[B55] Medina DD Kittrell FSF Hill JJ Shepard AA Thordarson GG Brown PP Tamoxifen inhibition of estrogen receptor-alpha-negative mouse mammary tumorigenesis Cancer Res 2005 65 3493 3496 15833886 10.1158/0008.5472.CAN-04-3869

[B56] Das R Vonderhaar BK Tamoxifen inhibits prolactin signal transduction in ER - NOG-8 mammary epithelial cells Cancer Lett 1997 116 41 46 10.1016/S0304-3835(97)04750-2 9177456

[B57] Oakes SRS Robertson FGF Kench JGJ Gardiner-Garden MM Wand MPM Green JEJ Ormandy CJC Loss of mammary epithelial prolactin receptor delays tumor formation by reducing cell proliferation in low-grade preinvasive lesions Oncogene 2007 26 543 553 10.1038/sj.onc.1209838 16862169

[B58] Sakamoto K Lin WC Triplett AA Wagner KU Targeting janus kinase 2 in her2/neu-expressing mammary cancer: implications for cancer prevention and therapy Cancer Res 2009 69 6642 6650 10.1158/0008-5472.CAN-09-0746 19638583 PMC2758773

[B59] Gonzalez-Suarez E Jacob AP Jones J Miller R Roudier-Meyer MP Erwert R Pinkas J Branstetter D Dougall WC RANK ligand mediates progestin-induced mammary epithelial proliferation and carcinogenesis Nature 2010 468 103 107 10.1038/nature09495 20881963

[B60] Wagner KU McAllister K Ward T Davis B Wiseman R Hennighausen L Spatial and temporal expression of the Cre gene under the control of the MMTV-LTR in different lines of transgenic mice Transgen Res 2001 10 545 553 10.1023/A:1013063514007 11817542

[B61] Andrechek ER Laing MA Girgis-Gabardo AA Siegel PM Cardiff RD Muller WJ Gene expression profiling of neu-induced mammary tumors from transgenic mice reveals genetic and morphological similarities to ErbB2-expressing human breast cancers Cancer Res 2003 63 4920 4926 12941816

[B62] Purdie CA Baker L Ashfield A Chatterjee S Jordan LB Quinlan P Adamson DJA Dewar JA Thompson AM Increased mortality in HER2 positive, oestrogen receptor positive invasive breast cancer: a population-based study Br J Cancer 2010 103 475 481 10.1038/sj.bjc.6605799 20664587 PMC2939790

[B63] Tworoger SS Hankinson SE Prolactin and breast cancer etiology: an epidemiologic perspective J Mammary Gland Biol Neoplasia 2008 13 41 53 10.1007/s10911-008-9063-y 18246319

[B64] Medina D Kittrell FS Shepard A Contreras A Rosen JM Lydon J Hormone dependence in premalignant mammary progression Cancer Res 2003 63 1067 1072 12615724

[B65] Mazumdar A Medina D Kittrell FS Zhang Y Hill JL Edwards D Bissonnette RP Brown P The combination of tamoxifen and the rexinoid LG100268 prevents ER-positive and ER-negative mammary tumors in P53-null mammary gland mice Cancer Prev Res (Phila) 2012 5 1195 1202 10.1158/1940-6207.CAPR-11-0524 22926341 PMC3837417

[B66] Yagi H Chuman Y Kozakai Y Imagawa T Takahashi Y Yoshimura F Tanino K Sakaguchi K A small molecule inhibitor of p53-inducible protein phosphatase PPM1D Bioorg Med Chem Lett 2012 22 729 732 10.1016/j.bmcl.2011.10.084 22115592

[B67] Natrajan R Lambros MB Rodriguez-Pinilla SM Moreno-Bueno G Tan DSP Marchio C Vatcheva R Rayter S Mahler-Araujo B Fulford LG Hungermann D Mackay A Grigoriadis A Fenwick K Tamber N Hardisson D Tutt A Palacios J Lord CJ Buerger H Ashworth A Reis-Filho JS Tiling path genomic profiling of grade 3 invasive ductal breast cancers Clin Cancer Res 2009 15 2711 2722 10.1158/1078-0432.CCR-08-1878 19318498

[B68] Howell A Bundred NJ Cuzick J Allred DC Clarke R Response and resistance to the endocrine prevention of breast cancer Adv Exp Med Biol 2008 617 201 211 10.1007/978-0-387-69080-3_19 18497044

