# Supplementary material for: Hormone-sensing cells require Wip1 for paracrine stimulation in normal and premalignant mammary epithelium
Source: Breast Cancer Res. 2013 Jan 31;15(1):R10. doi: 10.1186/bcr3381 (PMC3672744; doi:10.1186/bcr3381)
Supplement: Additional file 1 — Specifications for antibodies used in confocal immunofluorescence and fluorescence-activated cell sorting (FACS) analysis. [file bcr3381-S1.PDF]

Additional File 1 - Specifications for antibodies used in confocal immunofluorescence and FACS analysis

Antigen	Species	Dilution	Supplier	Cat#	Conjugate
Cytokeratin 8	Rat	1:100	Developmental Studies Hybridoma Bank	TROMA-1	
Estrogen Receptor	Rabbit	1:100	Santa Cruz Biotechnology	sc-542	
Estrogen Receptor	Mouse	1:100	Novacastra	NCL-ER-6F11	
phopsho-STAT5	Rabbit	1:300	Cell Signalling Technologies	9359	
phospho-p38	Rabbit	1:400	Cell Signalling Technologies	4511	
phospho-ERK	Rabbit	1:400	Cell Signalling Technologies	4370	
HER2	Rabbit	1:400	Cell Signalling Technologies	4290	
STAT5	Rabbit	1:200	Cell Signalling Technologies	9358	
Elf5	Goat	1:300	Santa Cruz Biotechnology	sc-9645	
Cytokeratin 14	Rabbit	1:4000	Covance	AF64	
CD45	Rat	1:500	Becton Dickinson	550994	PerCP-Cy5.5
CD24	Rat	1:250	Becton Dickinson	553261	FITC
CD49f	Rat	1:500	Becton Dickinson	555736	PE-Cy5
CD49b	Hamster	1:250	Becton Dickinson	558759	PE
Sca1	Rat	1:166	Becton Dickinson	558162	PE-Cy7
