# Supplementary figures and images for: Hormone-sensing cells require Wip1 for paracrine stimulation in normal and premalignant mammary epithelium
Source: Breast Cancer Res. 2013 Jan 31;15(1):R10. doi: 10.1186/bcr3381 (PMC3672744; doi:10.1186/bcr3381)

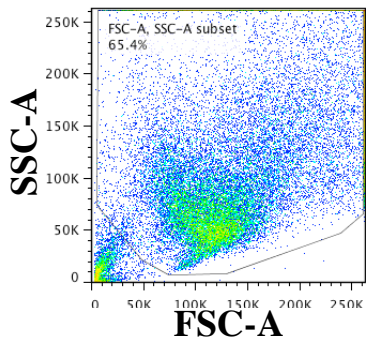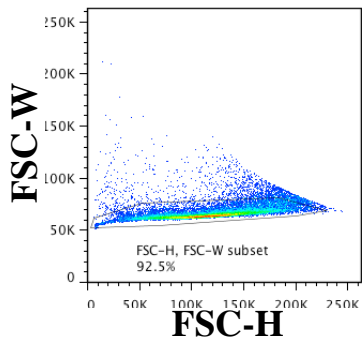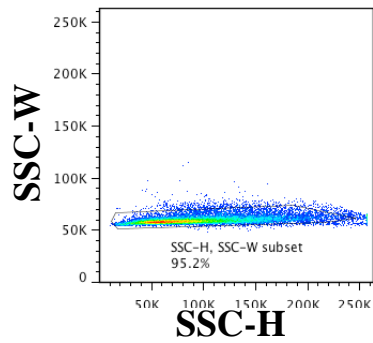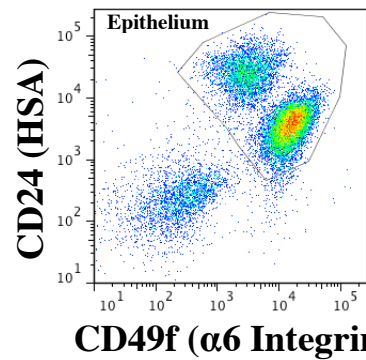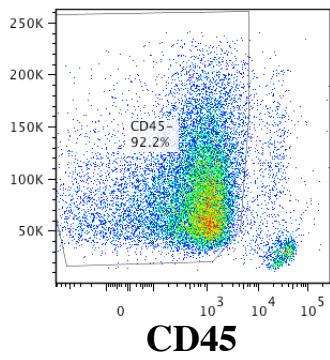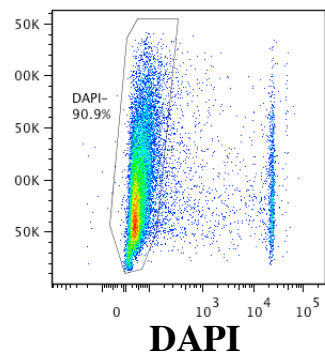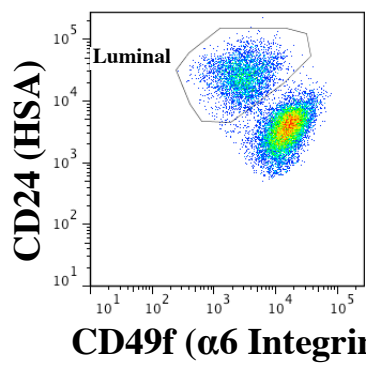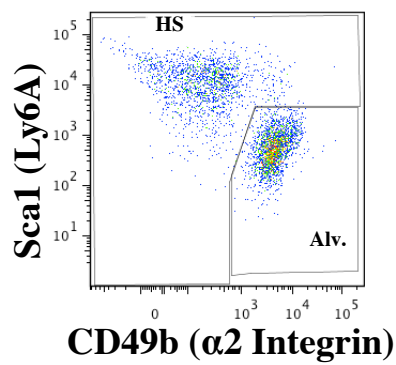

Supplement: Additional file 2 — Gating strategy used in all FACS analysis and sorting experiments. [file bcr3381-S2.PDF]

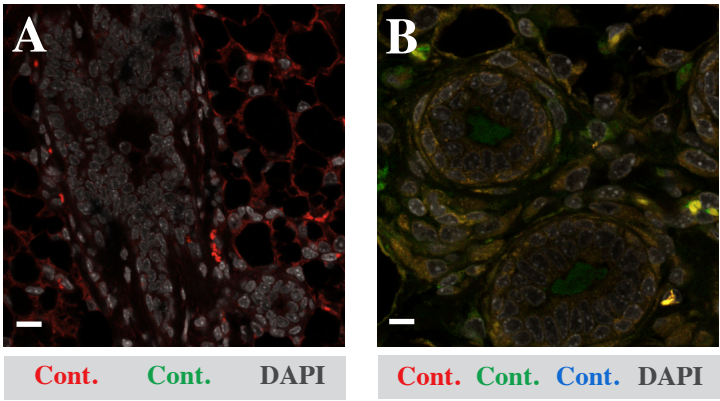

Supplement: Additional file 4 — Images of confocal immunofluorescence controls: sections of mammary tissue probed with goat-anti-mouse Alexa 488, goat-anti-rabbit Alexa 568 (A), and donkey anti-rat Alexa 633 (A, B) without the addition of primary antibody. [file bcr3381-S4.PDF]

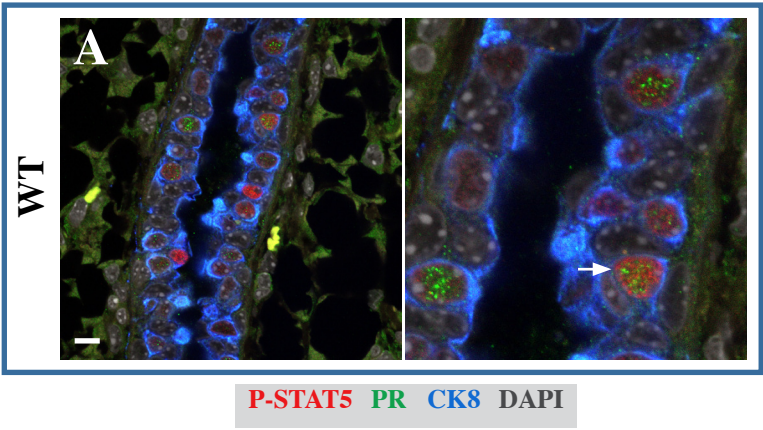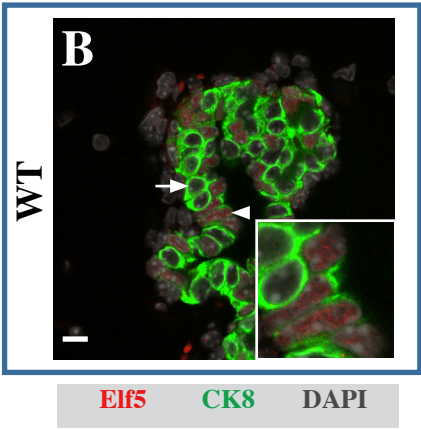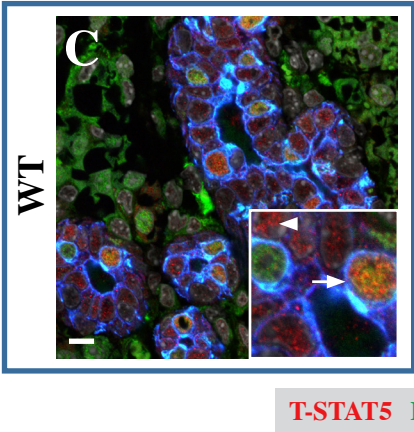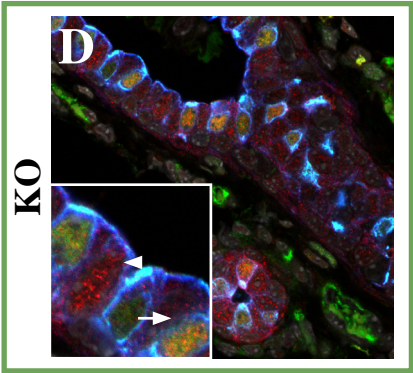

Supplement: Additional file 5 — Confocal immunofluorescence of mammary tissue section probed for progesterone receptor, total STAT5 and Elf5. Confocal immunofluorescence of mammary tissue from virgin wild-type mice probed for progesterone receptor & phosphorylated STAT5 (A) and Elf5 & cytokeratin 8 (B). Confocal immunofluorescence of mammary tissue from virgin wild-type (blue box) and Wip1 KO (green box) mice probed for total STAT5 and estrogen receptor (C, D). [file bcr3381-S5.PDF]

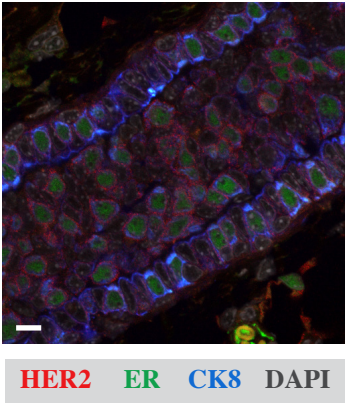

Supplement: Additional file 6 — Confocal immunofluorescence of virgin mouse mammary tumor virus (MMTV)-neu mammary tissue sections probed with antibodies specific for HER2/neu, estrogen receptor, and cytokeratin-8. [file bcr3381-S6.PDF]
