# Supplementary material for: Hormone-sensing cells require Wip1 for paracrine stimulation in normal and premalignant mammary epithelium
Source: Breast Cancer Res. 2013 Jan 31;15(1):R10. doi: 10.1186/bcr3381 (PMC3672744; doi:10.1186/bcr3381)
Supplement: Additional file 3 — Nucleic acid sequences for primers used in quantitative polymerase chain reaction (qPCR) experiments. [file bcr3381-S3.PDF]

Additional File 3 - Nucleic acid sequences for primers used in qPCR experiments

Mouse Gene ID	Accession Number	Sequence
HPRT (F)	NM_013556	CTGGTGAAAAGGACCTCTCG
HPRT (R)		TGAAGTACTCATTATAGTCAAGGGCA
Wip1 (Ppm1D) (F)	NM_016910.3	GGCAGCGTATGCTTCGGGCA
Wip1 (Ppm1D) (R)		TCCGGTGACTTGATTGGTGGTGT
Elf5 (F)	NM_010125.3	GGACTCCGTAACCCATAGCA
Elf5 (R)		TACTGGTCGCAGCAGAATTG
β-casein (F)	NM_009972	TCCACAACATTCCGTTTCTG
β-casein (R)		AGCATGATCCAAAGGTGAAAA
Estrogen Receptor (F)	NM_007956.4	GCCAAGGAGACTCGCTACTG
Estrogen Receptor (R)		CTCCGGTTCTTGTCAATGGT
Progesterone Receptor (F)	NM_008829.2	GGTGGAGGTCGTACAAGCAT
Progesterone Receptor (R)		CTCATGGGTCACCTGGAGTT
RANKL (F)	NM_011613.3	CCCACAATGTGTTGCAGTTC
RANKL (R)		TCCTGAGACTCCATGAAAACG
IGF-2 (F)	NM_010514.3	GTCGATGTTGGTGCTTCTCA
IGF-2 (R)		AAGCAGCACTCTTCCACGAT
Prolactin Receptor (F)	NM_011169.5	TTTTGCACATGAACCCTGAA
Prolactin Receptor (R)		ACCAGCAGGTGAATGTTTCC
